# Reliability and Validity Measures of the Patellofemoral Subscale KOOS-PF in Greek Patients with Patellofemoral Pain

**DOI:** 10.3390/jfmk10010044

**Published:** 2025-01-23

**Authors:** Ioannis Moros, Eleni C. Boutsikari, George Plakoutsis, Elefterios Paraskevopoulos, George A. Koumantakis, Maria Papandreou

**Affiliations:** 1 Laboratory of Advanced Physiotherapy, Department of Physiotherapy, University of West Attica, 12243 Athens, Greece; mscphys21008@uniwa.gr (I.M.); gkoumantakis@uniwa.gr (G.A.K.); 2 Department of Hygiene, Epidemiology and Medical Statistics, National and Kapodistrian University of Athens, 11528 Athens, Greece; e.boutsikari@med.uoa.gr; 3 School of Physical Education and Sports Science, National and Kapodistrian University of Athens, 17237 Dafne, Greece; elparaskevop@uniwa.gr

**Keywords:** patellofemoral pain, construct validity, test–retest reliability, KOOS-PF

## Abstract

**Background**: Patellofemoral pain (PFP) is one of the most common multifactorial musculoskeletal pathologies affecting the knee joint. The prevalence of PFP in the general population ranges from 11% to 17%, with higher rates observed in specific groups such as females, runners, military personnel, and young athletes. To assess symptoms associated with PFP, the patellofemoral subscale (KOOS-PF) was developed, consisting of 11 questions that evaluate pain, stiffness, and quality of life. The KOOS-PF scale has already been validated and shown to be reliable in both its Spanish and Arabic versions. **Objectives**: The aim of this study was to assess the reliability and validity of the KOOS-PF scale in the Greek language among the Greek population with patellofemoral pain. **Methods**: Fifty-five participants aged 18–65 years who suffered from PFP were evaluated in two phases on the first and third days to determine the reliability and validity of the measures of KOOS-PF in the Greek language. Construct validity was assessed using the knee outcome survey scale—activities of daily living scale (KOS-ADLS). Reliability was measured through repeated measurements (test–retest) using intraclass coefficient correlation (ICC), standard error of measurement (SEM), and smallest detectable difference (SDD). Internal consistency was evaluated using Cronbach’s coefficient a. The significance level was set at 5% (α = 0.05). **Results**: The KOOS-PF measures showed high internal consistency reliability (Cronbach’s alpha 0.87, *p* = 0.05) and high test–retest reliability (ICC = 0.95, *p* = 0.05, SEM = 3.7, SDC = 13.57). Additionally, the Greek version of the KOOS-PF exhibited high construct validity when correlated with the KOS-ADLS scale (r = 0.72, *p* = 0.001). **Conclusions**: The KOOS-PF scale displayed high reliability and construct validity for measuring patellofemoral pain in the Greek population.

## 1. Introduction

Patellofemoral pain (PFP) is one of the most common multifactorial musculoskeletal conditions. It is defined as pain originating from the patellofemoral joint or surrounding soft tissues in individuals, regardless of tissue damage [1]. PFP typically worsens during activities that stress the joint, such as deep squats, running, jumping, or climbing stairs [1,2,3].

The prevalence of PFP is significant, affecting approximately 23% of the general population and impacting specific groups, such as adolescent athletes, 25% of whom discontinue sport activities. PFP can lead to serious consequences, including musculoskeletal issues like osteoarthritis and a reduced quality of life, affecting the ability to perform daily activities [4,5]. PFP is more common in specific population groups, including females, runners, military personnel, and young athletes, negatively impacting not only musculoskeletal health but also overall quality of life, as described by the biopsychosocial model [1,6]. Furthermore, the primary cause of PFP might be a patellar trauma, though it is more commonly attributed to a combination of multiple factors (multifactorial causes), including overuse and excessive strain on the patellofemoral joint, anatomical or biomechanical irregularities, muscular weakness, imbalances, or dysfunctions [6].

Given the widespread impact of PFP, accurate assessment tools are essential for evaluating symptom severity, monitoring disease progression, and guiding appropriate treatment strategies [1,4,5,6]. Patient-reported outcome measures (PROMs) play a crucial role in understanding the functional limitations and quality of life impairments experienced by individuals with PFP [1,2]. These tools provide valuable insights into the patient’s perspective, allowing for a more comprehensive evaluation of their condition beyond clinical and imaging assessments. Among the available PROMs, the KOOS-PF scale has emerged as a highly relevant instrument, specifically designed to assess patellofemoral-related symptoms and functional impairments [7]. Its ability to detect subtle changes in symptom severity makes it particularly useful for both research and clinical practice, ensuring that interventions are appropriately tailored to the needs of individuals with PFP [7].

Such tools that evaluate patellofemoral pain (PFP) are the Kujala score, the patellofemoral pain syndrome severity scale (PPSSS) [7,8] and the patellofemoral subscale and osteoarthritis outcome score (KOOS-PF) [9]. However, the KOOS-PF is the most appropriate in mild cases of the disease and for its early detection and prevention in patients [10]. The KOOS-PF scale is widely recognized and has been validated in both Spanish and Arabic [10,11]. However, there is no validation of the KOOS-PF scale in Greek for the Greek population suffering from patellofemoral pain.

The aim of this study was to assess the reliability and validity of the KOOS-PF scale in Greek among the Greek population with Patellofemoral pain.

## 2. Materials and Methods

This study evaluated the validity and reliability of the patellofemoral scale (KOOS-PF) for the Greek population following the standard guidelines by [12]. It was approved by the Ethics Committee of the University of West Attica (No. 68323).

### 2.1. Participants

Fifty-nine (59) patients aged 18 to 65 years participated in this study, all diagnosed with patellofemoral pain. The inclusion criteria for the study were the following: (a) anterior knee pain diagnosed by the same orthopedic surgeon; (b) pain during functional activities in the patellofemoral joint, indicated by a VAS score > 5–6, which worsened with activities such as squatting, climbing/descending stairs, and running; (c) a duration of pain exceeding 3 months [9]. The exclusion criteria included the following: (a) pathological conditions such as stage 3 or higher knee osteoarthritis or recent surgeries (e.g., knee arthroplasty); (b) other pathological conditions, such as rheumatic diseases; (c) an inability to understand the Greek language.

### 2.2. Procedure

#### Translation

Permission was given by the main authors of the KOOS-PF subscale, Ref. [13], to translate it into Greek. The original version includes 11 self-assessment questions that are categorized in three groups: stiffness, pain, and quality of life. Every question has five possible answers. Each answer has a numeric value from 0 to 4. The final score is on a 0–100 scale, where 0 is the worst health score, and 100 represents the best score. The following steps were used in the translation:

1. Initial translation: a bilingual expert with knowledge of the medical terminology translated the KOOS-PF scale from English to Greek.
2. Back translation: another bilingual expert translated the Greek version back into English to check for consistency with the original text.
3. Expert review: a panel of researchers and clinicians reviewed both the Greek and the back-translated English versions to ensure that the meaning and context were preserved.
4. Pre-testing: the translated questionnaire was administered to a small group of Greek-speaking individuals to assess clarity and relevance.
5. Final adjustments: based on feedback from the pre-testing phase, necessary revisions were made to improve comprehension and cultural appropriateness.
6. Approval: the final Greek version was submitted to the main authors [13] for approval to ensure fidelity to the original subscale.

### 2.3. Outcome Measures

#### 2.3.1. Visual Analog Scale (VAS)

The VAS was administered to the patients during their demographic data collection to describe their pain experience [14].

#### 2.3.2. Patellofemoral Subscale (KOOS-PF)

The KOOS-PF subscale was administered twice to the patients, on the first and third days, to evaluate the reliability of the measurements [11,15,16]. The final score was calculated by taking the mean score of the 11 questions and dividing it by the maximum possible score for each question ranging from 0 to 100, where 0 indicates the worst health status, and 100 indicates the best one, using the following formula: Final score = 100 − [(average score ΕΠ1-ΕΠ11)/4 × 100].

#### 2.3.3. Knee Outcome Survey—Activities of Daily Living Scale (KOS-ADLS)

The KOS-ADLS was administered on the first day simultaneously with the KOOS-PF subscale to ensure construct validity of the KOOS-PF scale [15,16]. The KOS-ADLS was used with permission from the authors who had validated its use in the Greek language [17]. This scale consists of 14 questions that evaluate the symptoms of the knee joint pathology and the functional limitations experienced during daily activities.

### 2.4. Psychometric Assessment

#### 2.4.1. Reliability

Reliability was assessed through internal consistency, test–retest reliability, standard error of measurement (SEM), and smallest detectable difference (SDD) [18]. Internal consistency, evaluated through Cronbach’s alpha, was measured to assess whether the items were aligned with the same construct. Test–retest reliability was evaluated across time intervals and was quantified through the intraclass correlation coefficient (ICC). The standard error of measurement (SEM) assessed how precise the individual scores were while accounting for measurement error, and the smallest detectable difference (SDD) represented the smallest score difference that indicated an actual change rather than one due to measurement error [19].

#### 2.4.2. Construct Validity

The construct validity of the KOOS-PF scale was evaluated through convergent validity, which examined the degree of the KOOS-PF baseline scores when they were correlated with the KOS-ADLS scores using the correlation coefficient method [20].

### 2.5. Statistical Analysis

Data analysis was conducted using Stata 18.0 (Anon n.d.). For the description of the demographics and baseline data, we reported the mean value, the standard deviation (SD), the median value, the first (Q1) and third (Q3) quartiles, and the absolute and relative frequencies, as applicable. For the psychometric measurements, we reported the point estimates, the 95% confidence intervals (95% CI), and the respective *p*-values, as applicable. Precision was set to one decimal for the baseline and demographic data description, two decimals for the psychometric measurements, three decimals for the *p*-values, and four decimals for the factor analysis results. To be consistent with the benchmarks of the subject-to-item ratio method [21], we opted for a ratio of five participants per item [22]. Initially, exploratory data analysis (EDA) was conducted to explore the data distribution and characteristics [23]. The benchmark values and interpretation of Cronbach’s alpha [24], ICC [25], and correlation coefficient [26] are shown in Table 1. Regarding SEM and SDD, lower values indicate higher reliability [27]. Normality of the KOOS-PF, VAS, and KOS-ADLS was evaluated through the Shapiro–Wilk test [28] to select the appropriate correlation coefficient for quantifying the convergent validity of the KOOS-PF. Floor and ceiling (F/C) effects were categorized as significant (≥15%), moderate (10% to <15%), minor (5% to <10%), or negligible (<5%) [29]. The data were tested for suitability for factor analysis through inter-item correlations, the determinant of the correlation matrix, the Kaiser–Meyer–Olkin (KMO) statistic, and Bartlett’s test; inter-item correlations should lie between 0.30 and 0.90, the determinant was acceptable if greater than 0.00001, KMO values were acceptable if above 0.50, and Bartlett’s test was considered significant if the *p*-value was smaller than 0.50. To identify the number of factors to extract, both the Kaiser criterion (eigenvalues greater than 1) and the scree plot method were applied [30]. Factor loadings were evaluated based on thresholds of ≥0.32 for acceptable communalities, ≥0.5 for moderate communalities, and ≥0.8 for high communalities. A minimum of 60% variance was deemed acceptable to account for the total item variance [31]. The statistical significance was pre-specified at *p* < 0.05.

## 3. Results

Fifty-five (55) patients, primarily women (N = 31, 54.6%), with an established diagnosis of patellofemoral pain, aged from 18 to 65 years (mean age = 38.9, SD = 14.8), participated in the study. The participants’ average duration of patellofemoral pain was 7.5 (SD = 10.7) months, and the average pain intensity was 7/10 (SD = 0.9). The mean baseline KOOS-PF score was 36.3 (SD = 16.6). The EDA revealed no missing data across all variables. The demographics and baseline data are shown in Table 2.

### 3.1. Reliability

The Cronbach’s alpha point estimate was 0.87 (95% CI: 0.85 to 0.93, *p*-value < 0.001), the ICC was 0.95 (95% CI: 0.71 to 0.99, *p*-value < 0.001), the SEM was 3.7, and the SDD 13.6. All reliability measurements ranged from good to excellent, indicating a robust reliability of the Greek version of the KOOS-PF scale. The results are shown in Table 3.

### 3.2. Construct Validity

Construct validity was assessed by evaluating convergent validity between the baseline scores of the KOOS-PF, the VAS for pain [31], and the KOS-ADLS, since the KOOS-PF theoretically measures constructs related to those captured by the VAS and KOS-ADL. The correlations between the scales were analyzed through the appropriate correlation coefficient [20]. The validity assessment results demonstrated a high positive linear correlation between the KOOS-PF and the Greek KOS-ADLS scores (r = 0.72, 95% CI: 0.57 to 0.83, *p*-value < 0.001) and a moderate negative linear correlation between the KOOS-PF and the VAS scores (r = −0.64, 95% CI: −0.45 to −0.77, *p*-value < 0.001), supporting the construct validity of the KOOS-PF scale in this context. The results of construct validity are presented in Table 4.

### 3.3. Structural Validity

Factor analysis was used to model the relationships among the observed variables, using latent variables, and the factors. Exploratory factor analysis (EFA) was the method of choice, as no strong a priori hypothesis for the interrelations between the variables and the factors is available [32].

### 3.4. Factorial Validity

Table 5 reports the results of the inter-item correlation matrix. The KMO statistic was 0.826, Bartlett’s test of sphericity was significant (*n* < 0.001), and the determinant was 0.001, disproving multicollinearity. The lowest correlation was observed between items 1 and 8 (0.0529), while the highest was found between items 5 and 6 (0.8323). No correlations exceeded 0.9, but numerous were found below the lower threshold (0.3), primarily related to items 8 and 9. Given that the above-mentioned global tests indicated factorability, and that factor analysis was used on an exploratory basis in our research, we decided to proceed with an EFA, accommodating the varying inter-item correlations, through the principal axis factoring (PAF) model [33].

**Figure 1:**
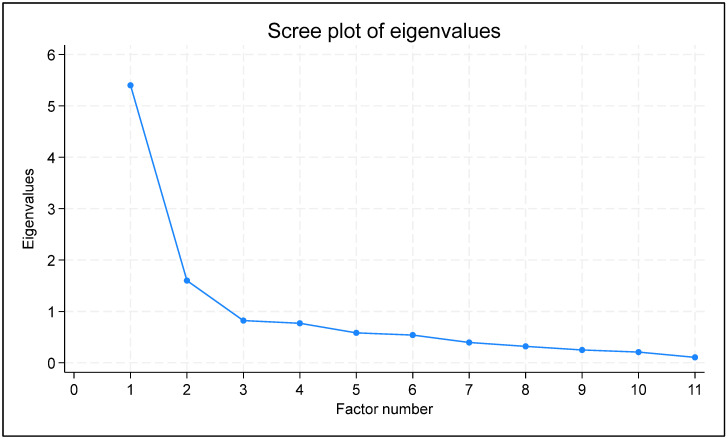
**Scree plot of eigenvalues.** .

The results of the PAF analysis are presented in Table 6. Two factors were retained, which explained 74% of the total variance: factor 1 explained 46.58% of the variance, and Factor 2 explained 27.42%. The moderate correlation (0.6103) between the factors suggests a meaningful but distinct relationship, supporting the use of oblique rotation [34]. Based on the Kaiser criterion and the scree plot (Figure 1), the two-factor model is the best representation of the 11-item Greek version of the KOOS-PF. The rotated factor loadings indicated that most variables related to everyday activities (items 1, 2, 3, 4, 5, 6, 10, 11) loaded highly on Factor 1. This suggests that Factor 1 could represent the construction of general physical function in everyday activities. In contrast, the two items related to jogging and running (items 8 and 9) had strong loadings on Factor 2, indicating that Factor 2 could capture a dimension of sport-related activities.

### 3.5. Floor and Ceiling Effects

The floor and ceiling (F/C) effects were evaluated through the percentage of respondents achieving the lowest (floor) or highest (ceiling) possible scores in a domain. High F/C effects, defined by a significant proportion of respondents scoring the lowest or highest possible values, indicate limited instrument range, measurement inaccuracy, and response bias, compromising the questionnaire’s performance. Historically, F/C effects above 15% are considered significant, though some recommend stricter thresholds for optimal sensitivity [29]. None of the subjects reached the worst or the best possible score; therefore, the KOOS-PF demonstrated no F/C effects. The results are summarized in Table 7.

## 4. Discussion

Based on recent clinical practice guidelines, the use of patient-reported outcome measures (PROMs) is largely important in the management of PFP, and reliable, valid tools should be used to achieve them [5]. The reliability measurements showed findings indicating scores ranging from above average to excellent, demonstrating a robust reliability of the Greek version of the KOOS-PF scale. Cronbach’s alpha point estimate was found to be 0.87, while the ICC was found to be 0.95, and the SEM and SDD values were low, indicating the high reliability of the KOOS-PF in Greek (Table 2). Evidently, all measurements pertaining to reliability were above average to excellent, indicating a robust reliability of the Greek version of the KOOS-PF scale.

These findings indicate the internal consistency of the KOOS-PF scale in Greek, ensuring almost similar painful situations, in terms of quality of life of Greek patients suffering from patellofemoral pain, as those of patients assessed with versions of the scale in other languages. In particular, our findings (Cronbach’s alpha 0.87) showed agreement of the Greek version with the original version (Cronbach’s alpha 0.86) [13] and the Arabic language version (Cronbach’s alpha 0.81) [21], while for the Spanish version, the internal consistency scores were the highest (Cronbach’s alpha 0.93) [11]. Overall, all the above results confirm the consistency of this scale when applied to different cultural populations.

The excellent reliability of the Greek version of the KOOS-PF describes the ability of the scale to perform accurately and consistently well. In the Greek and Arabic languages, the reliability of this scale was quite similar, and in these languages, the time estimate between repeated measurements was the same at 48 h; for the Spanish and the original versions, reliability was assessed after 1 and 2 weeks, also showing high values [11]. In addition, all the above studies showed lower SEM and SDD values as for the Greek version, indicating the clarity and precision of the questions contained in the KOOS-PF scale, ensuring a good understanding of the patients’ knowledge.

The construct validity of the KOOS-PF scale was assessed through convergent validity and showed a high positive linear correlation between KOOS-PF and KOS-ADLS scores (r = 0.72) and a moderate negative linear correlation between KOOS-PF and VAS scores (r = −0.64). These findings support the construct validity of the Greek version of the KOOS-PF scale in this context (Table 4). Therefore, the KOOS-PF did not show floor/ceiling effects for the patients’ scores, indicating no limited instrument range of measurement, measurement inaccuracy, and response bias that might compromise the performance of the Greek version of the KOOS-PF scale (Table 5).

Our findings are consistent with previous validity studies for the Spanish version (r = 0.71), the original version (r = 0.74), and the Arabic version, although the validity score was lower in these cases (r = 0.57) than ours [11]. Therefore, considering that the KOS-ADLS questionnaire is already validated for its reliability and validity, we considered that since the participants answered in almost the same way to similar questions in the two questionnaires, it means that they experienced what they described (pain, stiffness, quality reduction life) to the extent that they described them.

Regarding the results of the exploratory data analysis of the KOOS-PF scale, they indicated that there was insufficient sample adequacy, as the Kaiser–Meyer–Olkin (KMO) statistic was less than 0.70, to conduct confirmatory factor analysis [35,36]. According to Kaiser’s criterion [37], the selection of factors that could be used to explain variance was performed based on specific criteria, choosing factors that explained a significant percentage of variance (70–80% in total) and had an eigenvalue > 1, which is a very good value for use in statistical analysis (Figure 1). However, eight items (1, 2, 3, 4, 5, 6, 10, 11) showed high loading and communalities in the appropriate range (above 0.30). The decision to retain a single-factor solution was further supported by the scree plot. Subsequently, the above eight items were used for exploratory factor analysis, with a pre-hypothesized single factor. Loadings and item communalities of the eight items are presented in Table 6.

Regarding the factors mentioned above, indicating that most variables related to everyday activities (items 1, 2, 3, 4, 5, 6, 10, 11) loaded highly on Factor 1, it appeared that almost all questions of the KOOS-PF scale were either positively or negatively correlated with related activities, such as heavy household chores, jumping, running, and kneeling, along with corresponding symptoms like stiffness and pain during participation (Table 7). This suggests that Factor 1 could represent the construction of general physical function in everyday activities. The correlation of these factors indicated the patients’ self-efficacy in their actions and management.

In contrast, the two items related to jogging and running (items 8 and 9) had strong loadings on Factor 2, indicating that Factor 2 could capture a dimension of sport-related activities. This two-factor model supports the differentiation between general physical activities and more specific activities like jogging and running. The findings of this validity and reliability study will be further analyzed when the sample size of patients is larger, allowing for confirmatory factor analysis. Therefore, future studies should involve a larger sample size and specific populations, such as athletes, soldiers, and females, in Greece.

### 4.1. Limitations

One limitation of this study is the insufficient sample adequacy for confirmatory factor analysis, as indicated by the Kaiser–Meyer–Olkin (KMO) statistic being below 0.70. Additionally, the time interval differences in the reliability assessments (48 h for the Greek and Arabic versions vs. 1–2 weeks in other studies) may affect the comparability. However, it should be mentioned that based on previous research, a minimum sample of 55 participants can be considered “good” for the assessment of internal consistency, floor and ceiling effects, construct validity, test–retest reliability, and measurement error based on the recommendation of the consensus-based standards for the selection of health measurement instruments (COSMIN) [38]. It was advised to adhere to the recommendation of maintaining a minimum subject-to-item ratio of at least 5:1 for conducting exploratory factor analysis (EFA) and principal component analysis (PCA) [36]. Given that the final version of the KOOS-PF includes 11 items intended for factor analysis, it was recommended that the minimum study sample comprise at least 55 people.

### 4.2. Practical Applications

The findings of this study support the use of the Greek version of the KOOS-PF scale as a reliable and valid tool for assessing patellofemoral pain (PFP) in Greek-speaking populations. Given its strong internal consistency and test–retest reliability, clinicians and re-searchers can confidently implement this scale in both clinical practice and research settings to evaluate symptom severity, functional limitations, and treatment outcomes in patients with PFP. The high correlation of the KOOS-PF with other validated tools further reinforces its effectiveness in capturing the impact of PFP on daily activities and quality of life. Additionally, the absence of floor/ceiling effects ensures that the scale can accurately detect changes in patient-reported symptoms over time.

## 5. Conclusions

In conclusion, the KOOS-PF scale in Greek appears to be valid and reliable, providing clinical researchers with an effective evaluation tool for patients with patellofemoral pain.

## Figures and Tables

**Table 1:** Benchmarks and interpretation of Cronbach’s alpha, intraclass correlation coefficient, and correlation coefficients.

Statistic	Absolute Magnitude of the Observed Statistic	Interpretation
** Cronbach’s alpha **	0.01–0.06	Non-acceptable internal consistency
	0.61–0.70	Acceptable internal consistency
	0.71–0.80	Good and acceptable internal consistency
	0.81–0.90	Good internal consistency
	0.91–1.00	Excellent internal consistency
** Intraclass Correlation Coefficient **	0.00–0.49	Poor test–retest reliability
	0.50–0.75	Moderate test–retest reliability
	0.75–0.90	Good test–retest reliability
	0.91–1.00	Excellent test–retest reliability
** Correlation Coefficient **	0.00–0.10	Negligible correlation
	0.10–0.39	Weak correlation
	0.40–0.69	Moderate correlation
	0.70–0.89	High correlation
	0.90–1.00	Excellent correlation

**Table 2:** Demographics and baseline data (N = 55).

Variable (Continuous)	Mean Value (SD)	Median Value (Q1–Q3)	N
** Age (years) **	38.9 (14.8)	35.0 (26.0–55.0)	55
** Height (centimeters) **	172.5 (10.0)	171.0 (165.0–180.0)	55
** Weight (kilograms) **	76.7 (19.9)	70.0 (63.0–86.0)	55
** Body mass index (BMI) **	25.6 (5.3)	24.5 (22.2–27.3)	55
** Pain duration (months) **	7.5 (10.7)	4.0 (3.0–5.0)	55
** Pain intensity (VAS score) ^1^**	7.0 (0.9)	7.0 (6.0–8.0)	55
** KOOS-PF score ^2^**	36.3 (16.6)	30.1 (22.7–50.0)	55
** KOS-ADLS score ^3^**	50.6 (18.1)	47.1 (38.6–64.3)	55
** Variable (dichotomous) **	** Absolute Frequency **	** Relative Frequency **	55
** Gender assigned at birth **			
** Female **	31	56.4%	
** Male **	24	43.6%	

Notes: SD = standard deviation; Q1 = 1st quartile; Q3 = 3rd quartile; N = number of respondents; ^1^ Non-normally distributed variable (Shapiro–Wilk W statistic = 0.94, *p*-value = 0.013); ^2^ Normally distributed variable (Shapiro–Wilk W statistic = 0.97, *p*-value = 0.11); ^3^ Normally distributed variable (Shapiro–Wilk W statistic = 0.97, *p*-value = 0.23).

**Table 3:** Reliability measurements of the Greek version of the KOOS-PF scale.

Statistic	Point Estimate	95% CI	*p*-Value
Cronbach’s alpha	0.87	0.85 to 0.93	<0.001
Intraclass Correlation Coefficient	0.95	0.71 to 0.99	<0.001
Standard Error of Measurement (SEM)	3.7	-	-
Smallest Detectable Difference (SDD)	13.6	-	-

Notes: 95% CI = 95% confidence interval.

**Table 4:** Validity measurements of the Greek version of the KOOS-PF scale.

Correlation Coefficients	Point Estimate	95% CI	*p*-Value
KOOS-PF versus KOS-ADLS ^1^	0.72	0.57 to 0.83	<0.001
KOOS-PF versus VAS ^2^	−0.64	−0.45 to −0.77	<0.001

Notes: 95% CI = 95% confidence interval; ^1^ the correlation was assessed through Pearson’s correlation coefficient; ^2^ the correlation was assessed through Spearman’s correlation coefficient.

**Table 5:** Inter-item correlations of the Greek version of the KOOS-PF scale.

ITEMS	1	2	3	4	5	6	7	8	9	10	11
1	1.0000										
2	0.4308	1.0000									
3	0.3345	0.5710	1.0000								
4	0.4079	0.3871	0.5240	1.0000							
5	0.6000	0.4642	0.5785	0.5939	1.0000						
6	0.5838	0.5241	0.6206	0.6291	0.8323	1.0000					
7	0.3400	0.2739	0.3290	0.4740	0.4183	0.4050	1.0000				
8	0.0529	0.1129	0.2565	0.4381	0.3456	0.5449	0.3122	1.0000			
9	0.0739	0.1754	0.1949	0.3380	0.4226	0.4543	0.4342	0.6514	1.0000		
10	0.4454	0.5646	0.6306	0.5508	0.6176	0.6774	0.4376	0.3488	0.2928	1.0000	
11	0.4432	0.3205	0.5417	0.4075	0.5627	0.6267	0.2170	0.3997	0.2358	0.5142	1.0000

Note: This is a Spearman’s correlation matrix; Kaiser–Meyer–Olkin statistic = 0.826; determinant of the correlation matrix = 0.001; Bartlett test of sphericity chi-square = 331.879, *p*-value < 0.001.

**Table 6:** Summary of the exploratory factor analysis of the Greek version of the KOOS-PF scale.

	Factor 1	Factor 2
** Item 1 **	** 0.8220 **	−0.3115
** Item 2 **	** 0.6852 **	−0.1357
** Item 3 **	** 0.8573 **	−0.0988
** Item 4 **	** 0.5770 **	0.2652
** Item 5 **	** 0.7612 **	0.1691
** Item 6 **	** 0.7030 **	0.3539
** Item 7 **	0.3818	0.3816
** Item 8 **	−0.0152	** 0.9079 **
** Item 9 **	−0.0530	** 0.9216 **
** Item 10 **	** 0.7840 **	0.1131
** Item 11 **	** 0.7634 **	−0.0262
** Eigenvalue **	5.1241	3.0158
** % of variance **	46.58	27.42

Note: extraction method: principal axis factoring (PAF); rotation method: oblique rotation with Kaiser normalization; factor loadings >40 appear in bold.

**Table 7:** Floor and ceiling (F/C) effects of the KOOS-PF scale.

KOOS-PF Scale	KOOS-PF Value	% Scoring	Effect Classification
** Floor effects **	0 (minimum/worst score)	0%	negligible
** Ceiling effects **	100 (maximum/best score)	0%	negligible

## Data Availability

Dataset available on request from the corresponding author. The data are not publicly available due to privacy or ethical restrictions.
